# Depression and Its Association With Self-Esteem and Lifestyle Factors Among School-Going Adolescents in Kuala Lumpur, Malaysia

**DOI:** 10.3389/fpsyt.2022.913067

**Published:** 2022-06-09

**Authors:** Mohamad Faez Ibrahim, Wan Salwina Wan Ismail, Nik Ruzyanei Nik Jaafar, Ummi Kalthum Mohd Mokhtaruddin, Hooi Yee Ong, Nur Hidayah Abu Bakar, Hajar Mohd Salleh Sahimi

**Affiliations:** Department of Psychiatry, Faculty of Medicine, Universiti Kebangsaan Malaysia, Kuala Lumpur, Malaysia

**Keywords:** adolescent, south-east asia, eating disorder, internet addiction, physical activity

## Abstract

**Introduction:**

Depression is a prevalent mental health condition worldwide and in Malaysia. Depression among adolescents has been steadily increasing. Self-esteem has been known to be associated with depression. It has been postulated that a poor lifestyle among adolescents is associated with depression. This paper aims to study the correlation of self-esteem, lifestyle (eating behavior, physical activity, and internet usage) with depression among Malaysian youth.

**Methodology:**

This is a cross-sectional study among secondary school children from 5 random schools in an urban city of Kuala Lumpur, Malaysia. Those with intellectual disability and/or difficulty to comprehend Malay language, and without parental consent and assent, were excluded. Students from randomly selected classes aged 13-year-old to 17-year-old were invited to fill in these questionnaires: Socio-demographic Questionnaire, Rosenberg Self-esteem Questionnaire, Physical Activity Questionnaire (PAQ-A), Eating Disorder Examination Questionnaires (EDE-Q), Internet Addiction Test Scale (IAT), and Children's Depression Inventory (CDI).

**Result:**

461 students participated in the study. 21.5% of the participating students were found to have depression (*n* = 99). Younger age and Chinese race showed significant association with adolescent depression with a *p*-value of 0.032 and 0.017 respectively. Other significant correlations with depression were self-esteem (*p* = 0.013), disordered eating (*p* = 0.000), lower physical activity (*p* = 0.014) and problematic internet usage (*p* = 0.000).

**Discussion:**

The prevalence of depression among adolescents in this study (21.5%) is in line with previous prevalence studies in Malaysia. Self-esteem is postulated to be a moderating factor for depression hence explaining the significant association. A sedentary lifestyle may increase the risk of developing depression, The causal relationship between problematic internet usage and depression is complex and difficult to establish. This is similar to the relationship between problematic eating behavior and depression as well.

**Conclusion:**

There is still a need to explore the causal relationship between lifestyle factors and depression among youth. Despite that, the results from this paper have accentuated the gravity of the importance of a healthy lifestyle among adolescents. An appropriate preventive measure is governmental strategies and policies aiming at improving a healthier lifestyle in this age group.

## Introduction

Depression is characterized by persistent low mood, anhedonia, insomnia, fatigue, loss of appetite, feeling worthless, impaired concentration, and recurrent suicidal thoughts that are persistent and severe enough to cause impairment in function (1). Worldwide, depression is a common mental health problem and a leading cause of disability among adolescents. Approximately, 1.1% of adolescents aged 10–14 years, and 2.8% of adolescents aged 15–18 years are estimated to have depression (2). In America, 4.1 million adolescents experienced at least 1 episode of major depression (3). Adolescent depression is also prevalent in Malaysia, ranging from 18.3 to 32.7% (4–7) A large local survey involving 21,764 adolescents, found 16.6% of participants had mild depression, 12.8% had moderate depression whereas 3.8% had severe depression (8).

Several studies have examined adolescent depression in Malaysia, exploring different variables including sociodemographic factors, risky behavior and substance use, childhood adversities such as being bullied, and family-related factors (8–11). However, other important variables such as self-esteem and lifestyle factors are less frequently explored.

Self-esteem is defined as a stable sense of personal worth (12). It has been suggested that self-esteem was significantly lower among Asian adolescents (13) including adolescents in Malaysia (14). Notably, self-esteem has been significantly associated with adolescent depression (15–17) and reported as the strongest predictor of adolescent depression (16). It plays important role in mediating the relationship between loneliness and depression (18) and protects against suicidal ideation among Malaysian adolescents (19).

Unhealthy lifestyle factors such as physical inactivity, disordered eating, and excessive or pathological use of internet users have been shown to have significant relationships with adolescent depression.

The World Health Organization (WHO) Guideline 2020 defines physical inactivity as failure to complete at least 150 minutes of moderate physical activity or 75 min of vigorous physical activity or the combination of both intensities per week (20). According to the Adolescent Health Survey 2017, the prevalence of physically active adolescents in Malaysia was low (19.8%) as the majority did not participate inadequate physical activities. This is worrying since many studies reported an association between low physical activity and depression among adolescents. A longitudinal study found depression scores were lower among adolescents with persistently high levels of light activity. At age 12 and 14 years old, moderate to vigorous physical activity was negatively associated with depressive symptoms (21). Physical activity during early adolescence has also been found to predict lower levels of depressive symptoms in later years (22). Tajik et al. (23) investigated the relationship between physical activity and psychological correlates for example stress, depression, and anxiety, among 1,747 adolescents from the southern part of Malaysia. Their findings showed that level of physical activity was associated with anxiety and stress but not depression (23).

Disordered eating refers to unhealthy eating patterns with consequent negative psychological and physical effects (24). It is highly prevalent among adolescents in Malaysia (25) and the possible significant association with depression justifies special attention. An Iranian study found an association between disordered eating with adolescent depression, suggesting that very low self-esteem may be a shared correlate or risk factor for disordered eating and depression in Iranian adolescents and young adults (26). Two local studies observed a significant association between disordered eating and depression however the studies were conducted among University students instead of adolescents (27, 28). Conversely, a study among secondary school students in Malaysia, found a significant association with the emotional problems however it was not able to specify the type of emotional problems experienced (24).

The concept of internet addiction is an ongoing debate, hence is not currently recognized as a disorder in the World Health Organization (WHO), Diagnostic Statistical Manual of Mental Disorders−5 (DSM5), or International Classification of Disease−11 (ICD11) classifications (1, 29). In this study, internet addiction refers to the pattern of internet use by Young 2016, which was categorized into normal use i.e. “average online users” and pathological use i.e. “frequent problems due to internet usage” and “significant problems due to internet usage” (30).

The high internet penetration rate in Malaysia contributed to the increase in internet use over the past few years (24). According to the Internet Users Survey 2020, internet users among children and adolescents aged 5–17 years in Malaysia have increased from 28.5% in 2018, to 47% in 2020 (31) and 92% of adolescents have social media accounts (32). Alarmingly, awareness of parental control was low (31) despite the easy accessibility and increased use of the internet among adolescents. This new trend of internet use has strongly influenced the lifestyle of adolescents. Adolescent depression has been significantly associated with internet addiction (33, 34). Consistently, various local studies also reported a significant association between adolescent depression and internet addiction (35).

Adolescents comprise approximately 15.6 % of the total Malaysian population (36) Given that adolescence is a critical transitional period to adulthood with increased vulnerability for mental health problems, adolescent depressioneeds specific focus. Depression and its complication such as suicide, and social and academic impairment, among the large proportion of young people in the country, will potentially give a negative impact on the nation at large. Hence, understanding risk factors for adolescent depression is very important, allowing for early detection and treatment and consequently more favorable outcomes.

Self-esteem and lifestyle factors are important correlates of adolescent depression but were not adequately explored in Malaysia. This cross-sectional study aimed to determine the prevalence of depression and its associated factors such as socio-demographic factors, lifestyle factors, and self-esteem among adolescents attending secondary schools in Kuala Lumpur, Malaysia.

## Methodology

### Study Design, Setting, and Sample Population

A cross-sectional study was conducted in five randomly selected national secondary schools in the Federal Territory of Kuala Lumpur. Students from the randomly selected classes, aged 13 to 17 were invited to participate. Those with intellectual disability and/or difficulty to comprehend Malay language, and without parental consent and assent, were excluded. The study was conducted in mainstream public schools, indicated for students without learning disabilities, hence students with the stated issues would have been excluded. In isolated situations, teachers would screen the students to exclude those with learning and language issues. A total of 461 participants were required to complete the questionnaires during the given time in school. This study was approved by the Ministry of Education and department of education, Kuala Lumpur, Malaysia.

### Research Tools

#### Socio-Demographic Questionnaire

The demographic questionnaire consists of age, sex, race, religion, parental education, and marital status.

#### Rosenberg Self-Esteem Questionnaire

Rosenberg Self-esteem questionnaire (37) is a 10-item self-reported questionnaire used to assess global self-worth, including positive and negative feelings about the self. It is scored using a 5-point Likert scale from strongly agree to strongly disagree, with scores ranging between 10- and 50. The scores will be categorized as low (10–29), moderate (30–39), and high self-esteem (40–50). The questionnaire has been translated into Bahasa Malaysia and validated with good psychometric properties (38).

#### Physical Activity Questionnaire (PAQ-A)

The PAQ-A (39) is a self-administered questionnaire developed to assess general levels of physical activity for high school students aged 14 to 19 years of age. It provides a summary of physical activity scores derived from eight items, each scored on a 5-point scale. The mean of the 8 items is calculated and classified into low physical activity and high physical activity. The PAQ-A questionnaire was translated into Malay language and back-translated independently with permission by the authors.

#### Eating Disorder Examination Questionnaires (EDE-Q)

It is a 36-item (40) self-reported questionnaire assessing eating habits. It has four subscales i.e. eating concern, shape concern, weight concern, dietary restraint, as well as assessment for binge eating and compensatory behaviors. A higher score in the global and subscale denote more severe symptoms. The cut-off point for a global score of 4 is considered clinically significant. (41). Studies reported good internal consistency (42), test-retest reliability (43), convergent validity (42), and discriminative validity (44). The scoring will be measured quantitatively based on the result. EDE-Q had been translated to the Malay language and validated with good psychometric properties (45).

#### Internet Addiction Test Scale (IAT)

The pattern of internet usage among respondents was measured using the Internet Addiction Test Scale (IAT) (30), which is a 20-item self-reported scale. Items are scored using a Likert scale of 0–5 and categorized into “average online users” (i.e. score of 20–49), “frequent problems due to internet usage” (i.e. score of 50–79), and “significant problems due to internet usage” (i.e. score of 80–100). The higher the score depicts the more problematic the usage of the internet. IAT had been translated into Bahasa Malaysia and validated with good psychometric properties (46).

#### Children's Depression Inventory (CDI)

Depression among adolescents was assessed using the Children's Depression Inventory (CDI) (47). It is a 27-item self-reported scale assessing depressive symptoms in children and adolescents aged 7–17 years old. It has two scale scores and five subscale scores. Scale scores are for questions regarding the emotional problem and functional problem while the subscale scores are specified for negative mood/physical symptoms, negative self-esteem, interpersonal problems, anhedonia, and ineffectiveness. For each item, the respondent is presented with 3 choices from the absence of a symptom to a definite symptom that corresponds to three levels of symptomatology, ranging from 0-to 2. The higher the total score, the more severe the level of depression is. The total sum score of 20 was used as a screening cut-off score. (48) CDI has been translated to Bahasa Malaysia and validated with good psychometric properties (49).

### Ethical Issues

The study received ethical approval from Universiti Kebangsaan Malaysia (UKM) Research Ethics Committee (FF-2014-049), and approval from the Ministry of Education, Malaysia to approach schools for data collection.

### Statistical Analyses

Data analysis was done using the Package of Social Sciences (SPSS) for windows version 20. Descriptive statistics were reported for all variables. Correlation analyses were done using the Pearson chi-square test for categorical variables while the independent *t*-test was used for analysis between one categorical variable (depression) and one continuous variable (self-esteem). Yates's continuity correction was used for the correction of approximate error. Mann Whitney test was done to compare mean ranks between disordered eating/ physical activity and depression.

## Result

### Prevalence of Depression

21.5% of the participating students were found to have CDI total scores of above cut-offs for depression (*n* = 99).

### Socio-Demographic Characteristics of the Study Respondents

A total of 461 students participated in the study. Most of the participants were females (59.3%) and younger adolescents (62.5%). 66.8% were Malays (*n* = 308), 21.9% Chinese (*n* = 101), 9.8% Indian (*n* = 45) and 1.5% (*n* = 7) for other race, which is a representative of the Malaysian ethnic distribution. 67.5% of fathers and 71.4% of mothers had lower educational backgrounds, and most of the parents were married 89.5% (*n* = 413).

### Association Between Socio-Demographic Profiles and Adolescent Depression

Age and race showed significant association with adolescent depression. The younger adolescents aged 13–15 years (*N* = 71) were significantly more depressed compared to the older adolescents. In terms of ethnicity, 55.5% of the depressed adolescents were Malays, followed by Chinese (33.3%) and Indians (10%). Among the Chinese students, 33% were depressed compared to lower rates of 22 and 17% among the Malays and Indians respectively. (Table 1).

**Table 1:** Sociodemographic profiles and adolescent depression.

**Variables**	**Characteristics**	**Non depressed (*n* = 362) *N (*%)**	**Depressed (*n* = 99) *N (*%)**	**Tests**	***p*-value**
Age	13–15	217 (59.9)	71 (71.7)	4.596^a^	**0.032**
	16–17	145 (40.1)	28 (28.3)		
Sex	Male	140 (38.7)	48 (48.5)	2.705^b^	0.100
	Female	222 (61.3)	51 (51.5)		
Ethnic group	Malay	253 (69.9)	55 (55.6)	10.131^a^	**0.017**
	Chinese	68 (18.8)	33 (33.3)		
	Indian	35 (9.7)	10 (10)		
	Others	6 (1.6)	1 (1)		
Religion	Muslim	257 (71.0)	56 (56.6)		
	Buddha	63 (17.4)	35 (35.4)		
	Hindu Cristian	31 (8.6) 11 (3.0)	7 (7.1) 0 (0)		
	others	0 (0)	1 (1.0)		
**Socio-economic status (SES):** Father Mother	Low	242	69	1.151^a^	0.283
	High	99	21		
	Low	262	67	0.002^a^	0.965
	High	85	22		
Parents marital status	Married	328 (92.1)	85 (87.6)	1.923^a^	0.166
	Divorced	28 (7.9)	12 (12.4)		
	Others				

*^a^Pearson Chi-square*.

*^b^Yates continuity correction*.

*^c^Independent t-test*.

*Bold values signify statistical significance*.

### Association Between Self-Esteem and Adolescent Depression

Self-esteem and internet use were significantly associated with depression among adolescents. Low self-esteem was associated with 47.5% of depression whereas high self-esteem was associated with only 2% of depression among adolescents (Table 2).

**Table 2:** Association between self-esteem and adolescent depression.

**Variables**	**characteristics**	**Non depressed (*n* = 362) *N* (%)**	**Depressed (*n* = 99) *N* (%)**	**tests**	***p*-value**
Self-esteem	Low	34 (9.4)	47 (47.5)	2.488^c^	**0.013**
	Moderate	254 (70.6)	50 (50.5)		
	High	72 (20.0)	2 (2.0)		

*^a^Pearson Chi-square*.

*^b^Yates continuity correction*.

*^c^Independent t-test*.

*Bold values signify statistical significance*.

### Association Between Lifestyles and Adolescent Depression

Physical activity and disordered eating were significantly associated with depression. Physically active adolescents showed a significantly lower level of depression compared to less physically active adolescents. Adolescents with disordered eating were significantly associated with depression compared to those without. Depression was significantly associated with frequent use of the internet. Among the depressed adolescents, nearly half of them were moderate users of the internet (Table 3).

**Table 3:** Lifestyle and adolescent depression.

**Lifestyles i.e. eating patterns and physical activity**					
		**Lifestyle** **Mean rank (*****N*****)**		**Mann-Whitney test value**	***P*** **-value**
		Disordered eating	No disordered eating		
Depression		276.16 (*N* = 97)	213.61 (*N* = 356)	−4.172	**0.000**
		Low physical activity	High physical activity		
		175.89 (*N* = 91)	210.24 (*N* = 313)	−2.470	**0.014**
Lifestyle i.e. internet use					
Variables	Characteristics	Non depressed (*n* = 362) *N* (%)	Depressed (*n* = 99) *N* (%)	Tests	*p* value
Internet usage	Normal	102 (28.2)	14 (14.1)	30.165^a^	**0.000**
	Mild	162 (44.8)	31 (31.3)		
	Moderate	94 (26.0)	47 (47.5)		
	Severe	4 (1.1)	6 (6.1)		

*^a^Pearson Chi-square*.

*^b^Yates continuity correction*.

*^c^Independent t-test*.

*Bold values signify statistical significance*.

## Discussion

The current study found that 21.5% of the participants scored above the cut-off point for depression in the CDI questionnaire. This was akin to the prevalence reported by another local study using CDI (26.2%) in Kuching (the capital city of Sarawak, a state in Malaysia) (7). Other local studies (6, 8, 23, 50) also reported similar prevalence but the direct comparison was difficult given the different questionnaires used and the different locations where the studies were conducted.

Age and ethnicity were significant sociodemographic factors in depression among adolescents. The younger adolescents aged 13–15 years were significantly more depressed compared to older adolescents. This could be due to difficulties in adjusting to the new school environment and the lack of coping skills among the younger students. The combination of transitioning between primary school to secondary school and the onset of puberty may also be the cause of higher reports of depression in the younger age group in this sample (51). A 1-year longitudinal study in Australia reported that 32% of students transitioning from primary school to secondary school reported it being “difficult” (51). In this study, approximately half of the depressed adolescents were Malays, followed by Chinese and Indians. However, among the Chinese students, 33% were showing significantly more depressive symptoms compared to lower prevalence among the Malay and Indian adolescents. Few local studies had found significantly higher depression among Chinese adolescents (11, 52, 53), whereas another reported higher depression among Indian adolescents (8). A possible explanation, as described by Auerbach et al. is the presence of higher extrinsic aspiration (versus internal aspiration) among Chinese was linked to higher stress and depressive symptoms (54). It was also established that minority ethnic group has a higher rate of depression as compared to majority ethnic group (55, 56). In this study, the low number of Indian study respondents may be one of the reasons why this group did not show a similar relationship. Differences in races and ethnicities in Malaysia may be due to different cultural and religious practices, environmental and personal factors as well, for example, higher self-expectations and competitiveness (57). These ethnic differences need to be further explored and understood.

Consistent with previous findings (6, 15, 16), self-esteem was significantly associated with adolescent depression. Our finding showed that nearly half of the adolescents who reported high depressive symptoms reported low and moderate self-esteem, whereas only 2% had high self-esteem. Masselink et al. (58)suggested that self-esteem is a vulnerability factor in the development of depression in late adolescence, mediated independently by avoidance motivation and social problems. (58) Several theories attempt to explain the relationship between depression and self-esteem. The vulnerability model theory which proposes that poor self-esteem leads to depression has been reported to be robust and strongly supported (59). Another explanation of the relationship between self-esteem and depression is the “scar model” (depression eroding self-esteem) and also the diathesis-stress model (59).

Lifestyle factors such as internet use, physical activity, and disordered eating also showed significant association with adolescent depression. In our study, among the adolescents who reported high depressive symptoms, nearly half of them reported moderate use of the internet compared to only 26% in the non-depressed group. This was supported by previous studies reporting a significant association between internet use and depression among adolescents (60–62), but the causal relationship was unclear. The relationship between adolescent depression and problematic internet use is complex. Adolescents who were depressed may initially use the internet as a means of coping with the depressive symptoms, and the progression of untreated depression may cause anhedonia and poor energy and concentration which may reduce internet use eventually (63). On the contrary, an increase in internet use may cause social isolation, leading to depression (63).

In keeping with previous findings (64), this current study found a significant association between physical activity and depression (Table 3). Respondents with low physical activity reported more depressive symptoms. A recent cohort study of a large sample of adolescents found that a sedentary lifestyle at a younger age may increase the risk of developing depressive symptoms at 18 years of age (21). The mechanism is very complex. High physical fitness has been theorized to optimize the hormonal stress-responsive system, producing an anti-inflammatory effect and enhancing growth factor expression and neural plasticity (65), hence improving depressive symptoms, and vice versa.

Our findings showed adolescents with disordered eating were significantly associated with high CDI scores (depression) compared to those without. This is in line with previous studies where severe depression is associated with more severe eating behavior (66). Although some studies suggested self-esteem and depression as mediators for disordered eating (67), the link between depression and eating disorders is not yet clearly established (68). Adolescents who do have disordered eating may perceive body image disturbances which may lead to depression, on the other hand, adolescents who are depressed may also have disturbances in an eating pattern which can lead to an eating disorder.

This study contributes further to our understanding of adolescent depression in Kuala Lumpur, Malaysia, particularly the significant association of lifestyle factors with adolescent depression, which was not commonly explored in the local context. Nevertheless, findings need to be interpreted within the limitations of the study. The cross-sectional nature of the study does not allow causal interpretation of findings. Information on depression and its associated factors was based on self-reported questionnaires solely. The study was conducted in the urban area of Kuala Lumpur, hence may not be representative of Malaysian adolescents.

## Conclusion

In summary, this study reports that younger adolescents, adolescents with lower self-esteem, those having disordered eating, or lower physical activity and problematic internet usage are significantly associated with depression. Although a causal explanation was not established, it is noteworthy to highlight these relevant factors as a target for preventive measures by the Malaysian government for the adolescent age groups. Government strategies and policies promoting a safe healthy lifestyle among youth might be an appropriate approach.

## Data Availability Statement

The raw data supporting the conclusions of this article will be made available by the authors, without undue reservation.

## Ethics Statement

The studies involving human participants were reviewed and approved by Research and Ethics Committee UKM. The patients/participants provided their written informed consent to participate in this study.

## Author Contributions

MI, WW, and NN contributed to conception and design of the study. MI, UM, HO, and NA collected the data, organized the database, and performed the statistical analysis. MI, UM, HO, NA, WW, NN, and HM wrote sections of the manuscript. All authors contributed to manuscript revision, read, and approved the submitted version.

## Funding

Universiti Kebangsaan Malaysia Medical Centre (UKMMC) Fundamental Research Fund.

## Conflict of Interest

The authors declare that the research was conducted in the absence of any commercial or financial relationships that could be construed as a potential conflict of interest. The Handling Editor K-AT declared a shared affiliation, though no other collaboration, with one of the authors WW and NN at the time of the review.

## Publisher's Note

All claims expressed in this article are solely those of the authors and do not necessarily represent those of their affiliated organizations, or those of the publisher, the editors and the reviewers. Any product that may be evaluated in this article, or claim that may be made by its manufacturer, is not guaranteed or endorsed by the publisher.
